# DNA-Based Molecular Machines: Controlling Mechanisms and Biosensing Applications

**DOI:** 10.3390/bios14050236

**Published:** 2024-05-08

**Authors:** Chunran Ma, Shiquan Li, Yuqi Zeng, Yifan Lyu

**Affiliations:** 1 Molecular Science and Biomedicine Laboratory (MBL), State Key Laboratory of Chemo/Biosensing and Chemometrics, College of Chemistry and Chemical Engineering, Aptamer Engineering Center of Hunan Province, Hunan University, Changsha 410082, China; machunran@hnu.edu.cn (C.M.); shiquanli@hnu.edu.cn (S.L.); zyq000418@hnu.edu.cn (Y.Z.); 2 Furong Laboratory, Changsha 410082, China

**Keywords:** molecular machines, DNA nanotechnology, controlling mechanism, biosensing, multiplex detection, real-time monitoring

## Abstract

The rise of DNA nanotechnology has driven the development of DNA-based molecular machines, which are capable of performing specific operations and tasks at the nanoscale. Benefitting from the programmability of DNA molecules and the predictability of DNA hybridization and strand displacement, DNA-based molecular machines can be designed with various structures and dynamic behaviors and have been implemented for wide applications in the field of biosensing due to their unique advantages. This review summarizes the reported controlling mechanisms of DNA-based molecular machines and introduces biosensing applications of DNA-based molecular machines in amplified detection, multiplex detection, real-time monitoring, spatial recognition detection, and single-molecule detection of biomarkers. The challenges and future directions of DNA-based molecular machines in biosensing are also discussed.

## 1. Introduction

Molecular machines are molecule-based devices in which some components convert consumed energy into mechanical work. These biological molecular machines are ubiquitous within organisms and are integral to fundamental biological processes [1]. For instance, kinesins traverse along microtubule fibers, ATP synthase synthesize ATP through rotational mechanisms, and DNA replication machinery glides along DNA, among other examples [1,2]. Inspired by biological molecular machines, researchers have engineered a multitude of artificial molecular machines.

Since Feynman introduced the concept of nanotechnology [3], the artificial fabrication of nanoscale or microscale machines has become an increasingly appealing research field. Artificial molecular machines have made significant progress in the past few decades. The initial molecular machines were based on supramolecular chemistry. For example, the first prototype of the molecular machine was reported by Livoreil et al. [4] in 1994, which utilized the redox of copper ions to control the movement of two interlocked coordinating rings in [2]-catenate. Subsequently, a variety of molecular machines have been reported, such as molecular muscles, molecular elevators, molecular motors, and molecular cars [5,6,7,8,9,10,11,12]. Due to such remarkable achievements, the field of molecular machines was recognized with the Nobel Prize in Chemistry in 2016. Microscopic machines can achieve more precise operations at the nanoscale compared to macroscopic machines with visible mechanical movements. These artificial molecular machines have been developed for various biological applications, including killing cells [13,14], delivering chemicals [15], and guiding cellular behavior [16]. However, the construction and functionalization of molecular machines based on small molecules or supramolecular chemistry are limited by their inflexible molecular design and insufficient functional groups.

Benefiting from the high predictability, excellent programmability, easy synthesis and modification, and low immunogenicity, DNA has found wide applications in the fields of nanotechnology and biomedicine. In the 1980s, Seeman first demonstrated the potential of DNA molecules as building blocks in constructing various nanostructures [17,18,19,20]. Since then, DNA nanotechnology has emerged as a novel molecular engineering strategy, leading to the development of numerous DNA-based molecular machines (DMMs) such as tweezers, walkers, ratchets, and robots [21,22,23,24,25,26]. Compared with static DNA nanostructures with no mechanical motions, DMMs consume fuel and energy such as chemical energy, electric energy, and light energy [27,28,29,30] or respond to environmental changes to operate repetitively or continuously. Different controlling mechanisms endow DMMs with many unique structural and dynamic properties, which further enable their wide applications in the field of biosensing: (1) DMMs can accumulate weak target signals into strong output signal through dynamic and regenerable behavior, thereby achieving sensitive detection; (2) DMMs are able to integrate a growing array of recognition modules like aptamers and DNA enzymes [31,32,33,34,35], enabling the simultaneous detection of multiple biomarkers; (3) DMMs can dynamically respond to concentration changes in biotargets in living cells [36], enabling real-time in situ monitoring of biomarkers; (4) DMMs can recognize targets on cells or viral surfaces with accurate spatial distribution patterns through addressable structure design; (5) DMMs can be used to detect single-molecular targets by observing the dynamic structural transformation of DMMs at the single-molecule level.

**Scheme 1:**
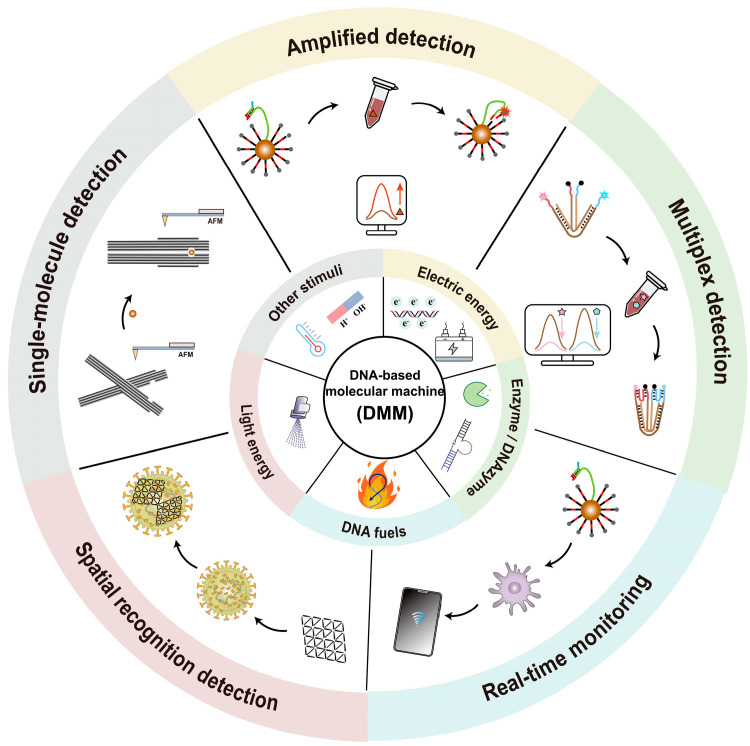
The controlling mechanisms and biosensing applications of DMMs.

This review focuses on the controlling mechanisms and biosensing applications of DMMs (Scheme 1). We first give a description of DMMs driven by different stimuli (such as DNA fuel, enzymes, electric fields, light, etc.). Unlike previous reviews that classify the applications of DMMs based on the type of targets, we discuss the applications of DMMs in amplified detection, multiplex detection, real-time monitoring, spatial recognition detection, and single-molecule detection based on the unique structural and dynamic properties of DMMs. Finally, we evaluate the future challenges and prospects in the development of DMMs.

## 2. The Controlling Mechanisms of DMMs

DMMs require input stimuli to achieve controlled operations. DNA fuel [37,38,39,40,41], enzyme [42,43,44,45,46,47], electric field [48,49,50,51], light [52,53], or other stimuli [54,55,56,57] have been used to trigger and maintain the operation of DMMs by providing necessary energy. Therefore, in this section, we classified DMMs based on the types of stimuli and introduced corresponding operation mechanisms.

### 2.1. DMMs Driven by DNA Fuels

**Figure 1:**
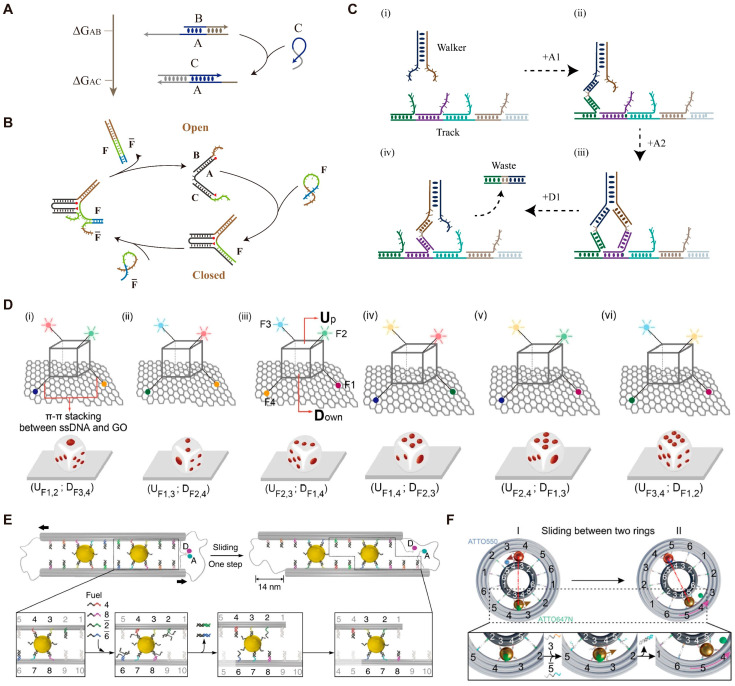
Examples of DMMs driven by DNA fuels. (**A**) Schematic diagram of DNA strand displacement reaction. (**B**) Schematic image of the DNA tweezer driven by F and $\overset{-}{F}$ strands. (**C**) Schematic image of the DNA walker driven by A1, A2, and D1. (**D**) Schematic images of the DNA nanodice on a GO platform. Reprinted with permission from [37]. Copyright 2023, Springer Nature. (**E**) Schematic images of the DNA sliding system. Reprinted with permission from [41]. Copyright 2018, Springer Nature. (**F**) Schematic images of the planetary gearset nanodevice. Reprinted with permission from [68]. Copyright 2022, American Chemical Society.

Single-stranded DNA can act as “fuel” to drive the operation of DMMs by forming new base pairs and releasing free energy (Figure 1A). The first DMM using DNA strands as fuel is called the DNA tweezer, as reported by Yurke et al. [58]. The DNA tweezer has two states, open state and closed state, as shown in Figure 1B. The alternating addition of fuel strand F and reset strand $\overset{-}{F}$ allows the repeat switch of DNA tweezer between open state and closed state. Similarly, Liu et al. [59] developed a DNA tweezer that can regulate the activity of glucose-6-phosphate dehydrogenase (G6pDH) by controlling the distance between the enzyme G6pDH and coenzyme NAD^+^. The introduction of fuel strands facilitates the approach of the enzyme and its cofactor, resulting in a 5-fold augmentation in enzymatic activity. Conversely, the introduction of a reset strand can inhibit the enzyme activity by opening the tweezer.

Another kind of DMM can move along the customized tracks and transport cargo from one site to another (Figure 1C). This kind of DMM was usually termed as a DNA walker or DNA molecular motor. A DNA walker usually includes a biped and a track, and is initially driven by DNA fuel-triggered strand displacement. [60] By adding DNA fuel strands, the movement of the walker along the track can be precisely controlled [24,61,62]. Inspired by the movement of bipedal motor proteins, in 2008, Yin et al. [63] reported an bipedal DNA walker that can unidirectionally move along a track based on a “burning bridge” strategy. After adding the fuel DNA strand, the front or rear foot of the bipedal DNA walker is randomly raised. Lifting the front foot allows the walker to move forward along the track, while lifting the rear foot causes the walker to fall off the track. This bipedal walker is unable to coordinate the lifting sequence of the front and back feet. In order to achieve coordination between the two feet, Omabegho et al. [64] improved the bipedal DNA walker system by ensuring that the movement of the front foot triggers the release of the rear foot. This ordered movement can help develop sophisticated molecular motor systems. DMMs can also move along other types of tracks besides DNA. For example, in 2023, Tang et al. [37] reported a DNA nanodice that can be thrown, manipulated, and cheated. The DNA dice consists of a skeleton made up of four DNA scaffold strands, with four different fluorophores connected to the vertices of the dice through single-stranded DNA (Figure 1D). Therefore, each face of the dice can be represented by different combinations of the fluorophores. The authors demonstrated that the dice can be randomly thrown on a graphene oxide (GO) platform (Figure 1D). The DNA dice adsorbed on the GO platform can be controlled to flip by adding DNA fuels. Furthermore, cheating can be achieved during the throwing process of the DNA dice by altering the bases composition, deleting bases, and adding chemical modifications.

The emergence of DNA origami provides a possibility for designing more complex DMMs. In 2015, Marras et al. [65] used DNA origami to design slider, hinge, crank-slider, and Bennett linkage, and drove the 1D, 2D, and 3D movement of these DMMs through strand displacement reactions. In the past few years, Liu’s research group has reported many DNA fuel-driven DMMs using origami [41,66,67]. For example, in 2018, Urban et al. [41] reported a DMM capable of relative sliding by crosslinking two DNA filaments in reverse parallel using two gold nanoparticles (Figure 1E). The ends of the filaments were connected by DNA hinges to ensure the reverse parallel arrangement. The addition of four DNA fuels triggered strand displacement reactions, resulting in the relative sliding of the DNA origami filaments. In 2019, Zhan et al. reported DNA origami-based nano gearset and controlled the coordinated movement of each component using DNA fuels [67]. Soon afterwards, Peil et al. [68] developed a DNA planetary gearset, in which strand displacement reaction triggered by DNA fuels provided the driving force, controlling the interaction and bidirectional rotation of the sun gear, planetary gear, and ring gear (Figure 1F).

### 2.2. DMMs Driven by Enzymes/DNAzymes

**Figure 2:**
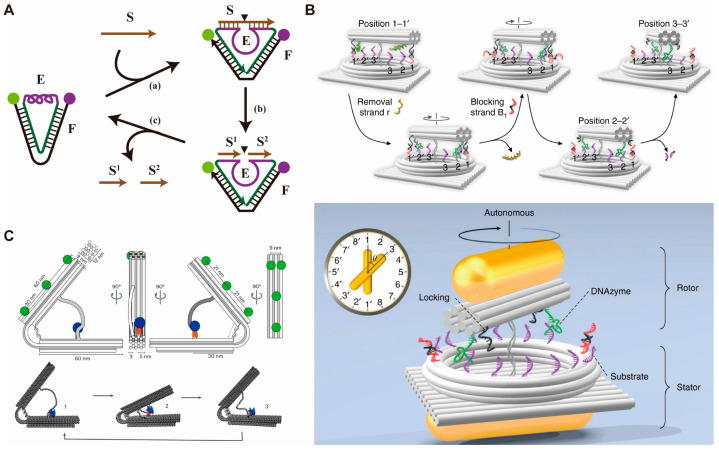
Examples of DMMs driven by enzymes/DNAzymes. (**A**) Schematic image of the DNA tweezer driven by 10–23 DNAzyme. (**B**) Schematic images of the DNA nanoclock driven by DNAzyme. Reprinted with permission from [71]. Copyright 2019, Springer Nature. (**C**) Schematic images of the DNA nanoengine driven by T7 RNA polymerase. Reprinted with permission from [45]. Copyright 2023, Springer Nature.

Because the speed of strand displacement reaction is relatively tardy, enzyme/DNAzyme-mediated strategies have been reported to accelerate the motion of DMMs [46,69,70]. In 2004, Chen et al. [46] reported a DNA tweezer by using 10–23 DNAzyme to hydrolyze the substrate strand (Figure 2A). Their DNA tweezer completes one open/close cycle every 1.5 min, which is much faster than the tweezers triggered by strand displacement reaction (about 10 min/cycle) [58]. The addition of the substrate strand can open the tweezers while cleavage of the substrate strand into two short strands by DNAzyme allow the DNA tweezers to return to the closed state and enter the next cycle. In the same year, Yin et al. [47] reported a DNA motor that can move unidirectionally along a track using T4 ligase and two restriction endonucleases. In this system, the walker is connected to the next anchorage by T4 ligase, and subsequently hydrolyzed from the previous anchorage by a restriction endonuclease. Since each connection site created can only be recognized by one type of specific restriction endonuclease, the movement of the walker is unidirectional. In 2010, Lund et al. [26] developed a molecular spider that moves randomly on a two-dimensional plane. The molecular spider consists of a streptavidin core, a single-stranded DNA, and three 8–17 DNAzymes-based legs. The DNAzyme-mediated cleavage of DNA anchor strands allows the movement of the molecular spider. Unlike previously reported DNA walkers that move along a one-dimensional track, the molecular spider can achieve Brownian walks on a two-dimensional plane and move up to 100 nm (approximately 50 steps). Moreover, Qu et al. [69] reported a DNA walker with autonomous movement on the three-dimensional surface of gold nanoparticles, which is driven by exonuclease III-mediated DNA hydrolysis. In 2019, Xin et al. [71] designed a DNA plasmonic nanoclock that can autonomously rotate along an origami-based circular track (Figure 2B). The rotor is composed of gold nanorods connected to DNA origami sticks containing two DNAzyme-based feet, while the stator consists of another gold nanorod connected to a DNA origami platform. A circular track includes 16 anchoring strands. DNAzyme catalyzes the cleavage of the anchoring strands to drive the rotation of the DNA clock.

To further accelerate the speed of DMMs, Yehl et al. [70] developed a DNA rolling motor by modifying single-stranded DNA on the surface of silicon dioxide nanoparticles. Hydrolysis of RNA tracks by RNase H propelled the rolling of nanoparticles. The speed of the rolling motor can reach up to about 5 μm min^−1^, which is approximately 5000 times faster than the previously reported DNA walker. In addition to nucleases that catalyze DNA/RNA hydrolysis, DMMs can also be driven by polymerase [43,44]. In 2023, Centola et al. [45] introduced the T7 RNA polymerase (T7 RNAP)-driven DNA leaf-spring nanoengine with two opposing origami arms connected by a DNA hinge and a double-stranded DNA template linker (Figure 2C). A T7 RNAP was connected to a DNA arm through HaloTag. Upon transcription along the double-stranded DNA template, the T7RNAP pulls the opposing origami arm towards itself, forcing the structure to close. When the terminator sequence is reached, the T7RNAP releases the dsDNA template, allowing the nanoengine to open.

### 2.3. DMMs Driven by Electric Field

**Figure 3:**
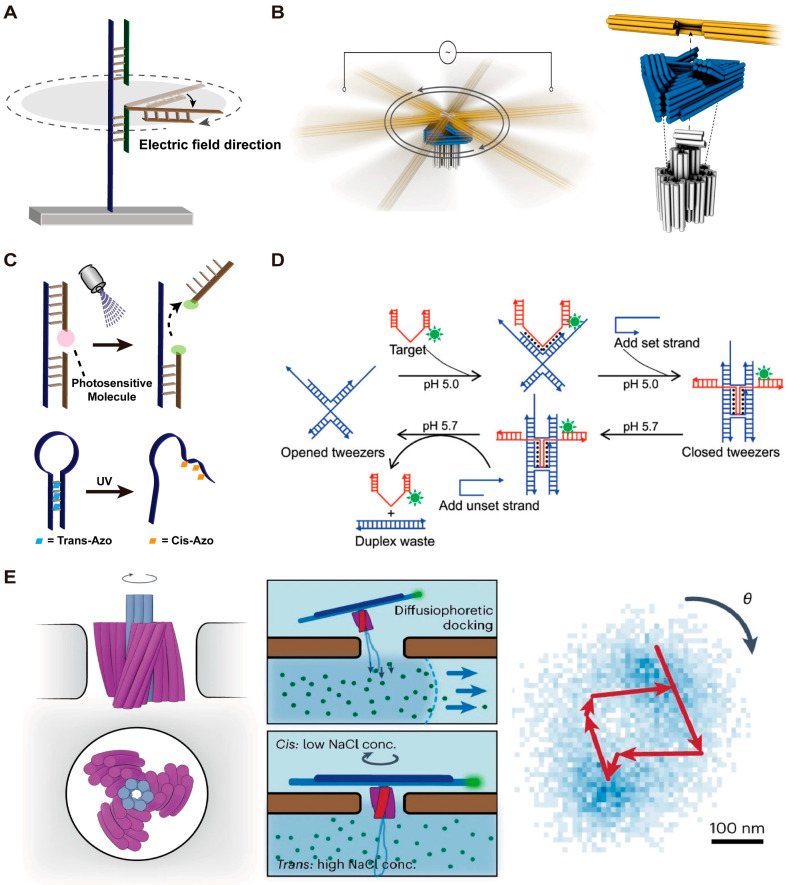
Examples of DMMs driven by electric field and other stimuli. (**A**) Schematic images of the DMMs driven by electric field. (**B**) Schematic images of the DNA ratchet driven by electric field. Reprinted with permission from [49]. Copyright 2022, Springer Nature. (**C**) Schematic images of the DMMs driven by light. (**D**) Schematic images of the DNA tweezer driven by PH value. Reprinted with permission from [75]. Copyright 2008, American Chemical Society. (**E**) Schematic images of the DNA turbine driven by ion concentration gradient. Reprinted with permission from [50]. Copyright 2023, Springer Nature.

The high-speed operation of macroscopic machines driven by electric fields inspired the notion that negatively charged DMMs might be also able to operate at a faster speed with the help of an electric field (Figure 3A). In 2018, Kopperger et al. [48] first used an electric field to precisely control the movement of a DNA motor. The DNA motor consists of a 55 nm square DNA origami platform and a 25 nm nanorobotic arm and is fixed at the bottom of a cross-shaped electrophoretic chamber filled with buffer. Due to the strong force generated by the alternative electric field, this DNA motor can rotate with a speed at least five orders of magnitude faster than previously reported DNA motor systems and can even propel a gold nanoparticle. In 2022, Pumm et al. [49] developed a ratchet molecular motor based on DNA origami with rotational speeds and torques under an alternating electric field that are comparable to those of the natural molecular motor F_1_F_0_-ATPase (Figure 3B). They also utilized a variant of this machine to store elastic potential energy. When the alternating electric field is turned off, the release of elastic potential energy stored in a single-stranded DNA loop causes the ratchet to rotate in the opposite direction (Figure 3B). In 2023, Vogt et al. [51] used a molecular joint to connect the nanorobotic arm with a base plate and constructed a torsion spring, which was further used to store and release the mechanical energy.

### 2.4. DMMs Driven by Light

Light can also serve as stimuli to trigger the motion of DMMs (Figure 3C). Typically, the light energy is first absorbed by photosensitive molecules, then converted into chemical energy by causing bond broken or strand dissociation. For example, previous work has reported that pyrene molecules can catalyze the cleavage of disulfide bonds in the DNA phosphate backbone under ultraviolet light [72]. Based on this, in 2012, You et al. [29] developed a light-controlled DNA walker. The DNA walker consists of a 16 nt long leg and a 7nt short leg and is modified with pyrene molecule. Each anchor strand on the track contains a disulfide bond in the middle. When exposed to 350 nm light, pyrene molecules can promote the cleavage of disulfide bonds, leading to the DNA walker taking a step forward. In the same year, You et al. [73] reported a DNA molecular motor that can move along a track in different directions controlled by light of different wavelengths. This strategy is achieved by modifying the anchor strands on the track with azobenzene molecules. Light of different wavelengths can cause cis/trans conformational changes in azobenzene molecules. Cis-azobenzene can inhibit DNA hybridization while trans-azobenzene can stabilize the DNA hybridization. Therefore, light of different wavelengths can induce changes in the affinity between the anchor strands on the track and the DNA walker, thereby controlling the moving direction of the DNA walker. Subsequently, Skugor et al. [74] developed a light-controlled and non-autonomous DNA walker. The two types of azobenzene derivatives were controlled by orthogonal wavelengths, enabling precise control over the movement and position of the DNA walker at each step.

In 2018, Liu et al. [21] reported a DNA nanotweezer that can transform from a closed state to an open state in response to ultraviolet light. The nanotweezer consists of a poly-A hinge and two DNA tile arms. The trigger strand poly-T is connected to the DNA tweezer and modified with 6-nitropiperonyloxymethyl (NPOM) groups as a photocage protecting group. The tweezer is in a closed state initially, and the NPOM groups are detached when exposed to UV light at a wavelength of 365 nm. Therefore, the trigger strand can bind to the hinge domain, causing the DNA tweezer to open. The opening speed of the tweezers using this strategy is approximately 60 times faster than the previously reported DNA tweezer triggered by strand displacement reaction [59].

### 2.5. DMMs Driven by Other Stimuli

Apart from the stimuli discussed above, there are other external stimuli that have been reported to provide energy for DMMs, such as pH, temperature and concentration gradient. Hydrogen ions can protonate the N3 position of cytosine, and the protonated cytosine can then form Hoogsteen hydrogen bonds with guanine [76], therefore inducing the formation of the DNA triplex. In 2008, Han et al. [75] successfully used DNA tweezers to precisely capture and release a DNA cargo by changing its pH value. Specifically, at pH 5.0, the DNA cargo can bind to the DNA tweezer through Hoogsteen hydrogen, and the set strand is added to immobilize the captured cargo (Figure 3D). By changing the pH to 5.7 and adding the unset strand, the DNA tweezer is opened, and the cargo is released (Figure 3D). Moreover, in 2020, Thompson et al. [77] developed a universal molecular switch for controlling the activity of aptamers by regulating the pH value.

Concentration gradients can also be used to drive the motion of DMMs. Inspired by the working mechanism of F_0_F_1_-ATP synthases, in 2023, Shi et al. [50] developed a DNA origami-assembled turbine, which can rotate when there is an ion concentration gradient at both ends (Figure 3E).

Gerling et al. [57] demonstrated that complex DMMs could be constructed using shape-complementary approaches, which is inspired by ribonuclease P (RNase P). This method does not rely on base pairing, but rather connects the components of molecular machines through weak interactions involving nucleobase stacking, just like building “Lego” blocks. Using this method, the authors developed a robot with moving arms, a book that can be opened and closed, a gear transmission device that can be switched on and off, and a DNA switch. The assembly, disassembly, and movement of these molecular machines can be controlled by changing the temperature or cation concentration.

## 3. Biosensing Applications of DMMs

Benefitting from advantages such as biocompatibility, programmability, easy functionalization, and stimulus-controlled motion, DMMs have been widely applied in the field of biosensing. In this section, we introduce the biological sensing applications of DMMs from five aspects: amplified detection, multiplex detection, real-time monitoring, spatial recognition detection, and single-molecule detection.

### 3.1. DMMs for Amplified Detection of Biomarkers

Sensitive detection of biomolecules is crucial for elucidating the molecular mechanisms of life processes and accurately diagnosing diseases. Due to the low abundance of certain biomarkers in biological environments, it is necessary to develop signal amplification strategies to increase the sensitivity of the detection process [78,79].

**Figure 4:**
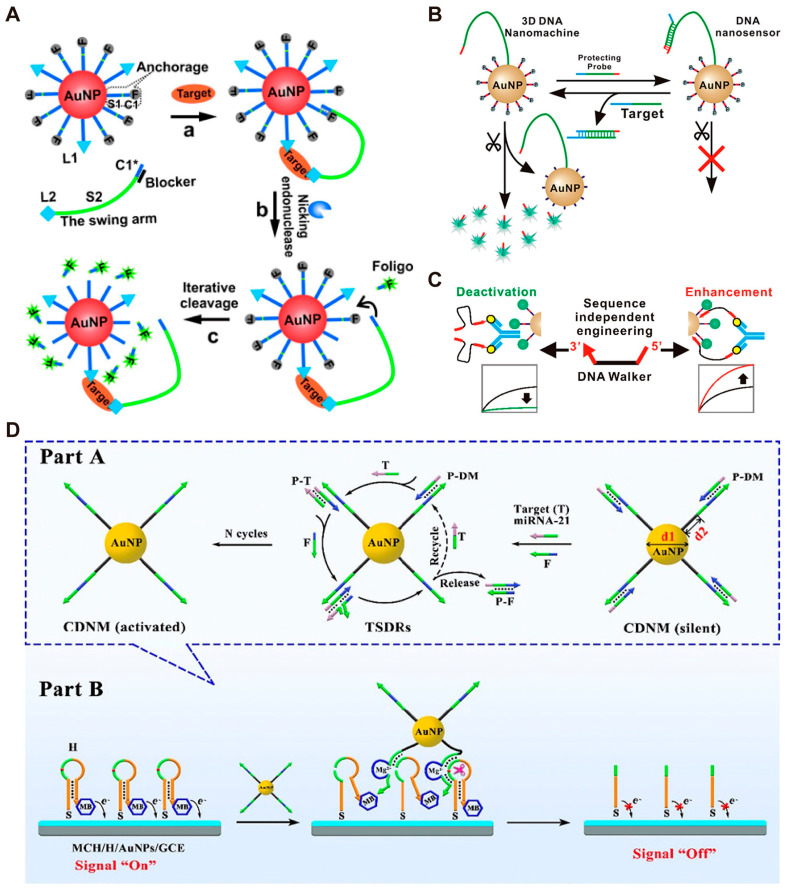
Examples of DMMs for signal amplification. (**A**) Schematic image of binding-induced 3D DNA walker. Reprinted with permission from [85]. Copyright 2015, WILEY—VCH Verlag GmbH & Co. KGaA, Weinheim. (**B**) Schematic image of the DNA walker containing protecting probe. Reprinted with permission from [86]. Copyright 2016, American Chemical Society. (**C**) Schematic image of adjusting DNA walker’s interface factors. Reprinted with permission from [88]. Copyright 2021, American Chemical Society. (**D**) Schematic images of the preparation and walking process of the CDNM. Reprinted with permission from [90]. Copyright 2023, American Chemical Society.

It is worth noting that the DNA walker itself could serve as a signal amplifier [42,80,81,82,83,84]. The three-dimensional (3D) DNA walker constructed by Zhang et al. [85] is a typical example. They immobilized both DNA track strands and ligand strands on the surface of AuNPs. The ligand strand on AuNP surface can capture the target and form a sandwich structure with the walking strand so that the walking strand can be immobilized on the nanoparticle surface (Figure 4A). Driven by nicking endonuclease, the walking strand continuously moves around AuNP surface and cleaves multiple track strands, eventually leading to amplification of fluorescent signals. Yang et al. [86] also utilized AuNPs to construct a 3D DNA walker by combining strand displacement reaction with amplified detection of target nucleic acids and achieved a limit of detection (LOD) lower than 20 pM. (Figure 4B). A similar strategy has been employed for detecting nucleic acid in Ebola virus with an LOD of 3.5 fM [87]. In addition to nucleic acid detection, Yang et al. [88] designed a set of dual-foot DNA walkers for detecting antibodies by modifying small molecule ligands at the 3′ end or both the 3′ and 5′ ends of the walking strand. They demonstrated that the sensitivity of the walker in complex sample matrices such as human serum samples could be enhanced by adjusting interface factors such as orientation, cooperativity, and spatial effects (Figure 4C). Recently, Zhang et al. [89] developed DMMs utilizing quantum dots and aptamers for sensitive detection of circulating tumor cells in human blood, with a detection limit of 2 cells/mL. DMMs can be used to amplify not only fluorescent signals but also electrochemical signals. Recently, Li et al. [90] developed an efficient and controllable three-dimensional DNA nanomachine (CDNM) for miRNA-21 detection. Compared to traditional DNA nanomachines, CDNM showed higher amplification efficiency and faster walking speed by adjusting the diameter of the core (AuNPs) and the length of the walking strand (DNAzyme), with an LOD as low as 33.1 aM (Figure 4D). Unlike strategies that require modification, Williamson et al. [91] utilized DNA origami-based DMMs to construct label-free electrochemical biosensors, achieving the amplification of electrochemical signal. Liu et al. [92] developed an aptamer-based DNA rolling motor for adenosine detection with an LOD of 0.17 nM.

**Figure 5:**
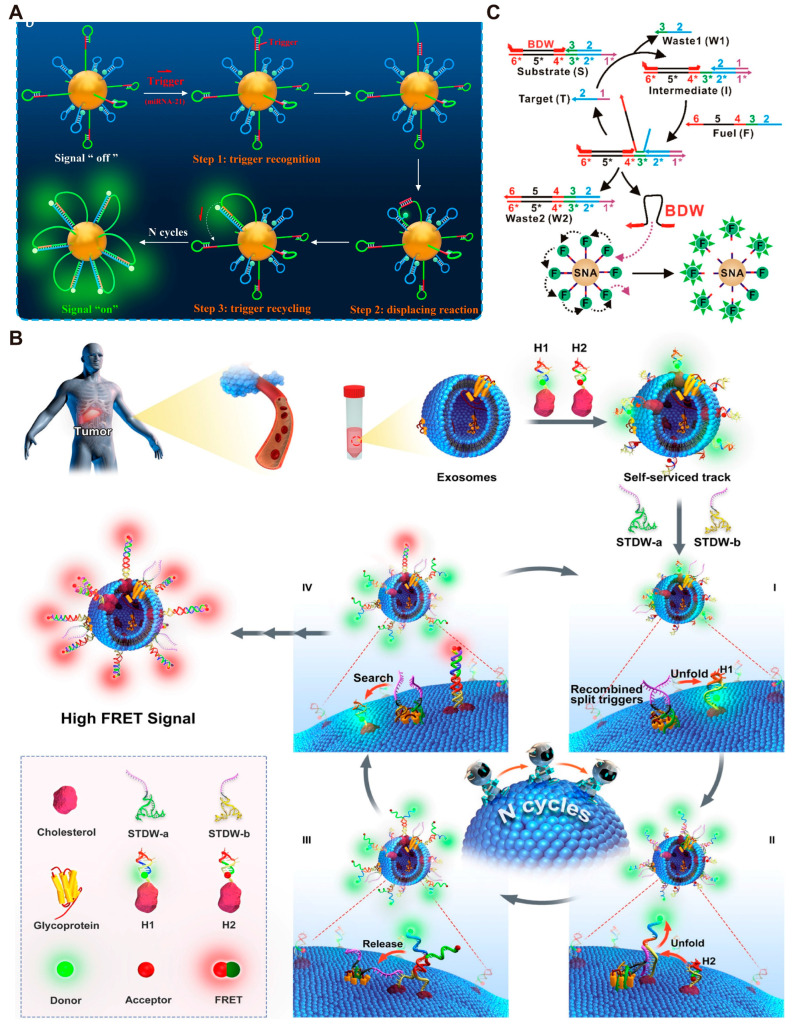
Examples of DMMs coupled with other signal amplification strategies. (**A**) Schematic images of the preparation and walking process of UDDNM. Reprinted with permission from [103]. Copyright 2023, Elsevier B.V. (**B**) Schematic image of STDW. Reprinted with permission from [23]. Copyright 2022, Wiley-VCH GmbH. (**C**) Schematic image of target-specific activation of BDW using CHA. Reprinted with permission from [105]. Copyright 2019, American Chemical Society.

DMMs can also be coupled with various enzyme-free signal amplification strategies, including catalytic hairpin assembly (CHA) [93,94] and hybridization chain reaction (HCR) [95,96,97], to further improve detection sensitivity [61,98,99]. CHA is an enzyme-free, efficient isothermal amplification reaction. This reaction can be activated by a specific single-stranded DNA (ssDNA) to catalyze the self-assembly of two substrates (DNA hairpins), resulting in the generation of a large amount of double-stranded DNA (dsDNA) [100,101,102]. Recently, Meng et al. [103] coupled DNA walkers with CHA to construct an ultrafast dual-layer 3D DNA nanomachine (UDDNM). Specifically, the target (miRNA-21), acting as a catalyst in the CHA reaction, hybridizes with the outer long hairpin 1 (H1). Then H1 hybridizes with inner short hairpin 2 (H2), resulting in fluorescence signals (Figure 5A). The construction of the double-layered structure can increase the effective collision between the target and probe H1 and reduce the steric hindrance of the reaction. Therefore, UDDNM was successfully used for ultra-sensitive fluorescence detection of miRNA-21 and sensitive intracellular imaging. Apart from AuNPs, Wang et al. [23] utilized the tumor-derived exosomes (Exos) as the 3D track to construct a self-serviced-track DNA walker (STDW). Once the split aptamer binds to the target (the glycoprotein on exosomes), the autonomous movement of the walker is initiated based on CHA (Figure 5B). Using this method, tumor-derived exosomes in cell culture media and serums can be directly detected without washing steps. Entropy-driven amplification (EDA) reaction is another enzyme-free amplification strategy, initially proposed by Zhang et al. in 2007 [100]. Compared to CHA, this strategy does not require the hairpin structure, resulting in fewer sequence constraints [104]. Mason et al. built a dual signal amplification system with the detection limit of 10 fM for nucleic acid targets by combining EDA with the 3D DNA walker [105]. In their design, the target can be recycled in the EDA reaction, generating a large number of bipedal walking chains (BDW) for the next step of walking amplification (Figure 5C). Additionally, EDA can decouple the target sequence from the sequence of the bipedal DNA walker. Therefore, this versatile strategy can be generalized to any target sequence without altering the design of BDW.

In addition to enzyme-free amplification strategies, DNA walkers can also be integrated with enzyme-assisted amplification strategies [106]. RCA is a common enzyme-assisted isothermal nucleic acid amplification method, suitable for both signal amplification and target amplification [107]. In an RCA reaction, DNA or RNA is used as a primer to synthesize long ssDNA using a circular ssDNA template with the assistance of a polymerase [100]. Zheng et al. [108] devised a nanostructure that integrates DNA walkers and RCA for ultra-sensitive detection of human immunodeficiency virus (HIV). Assisted by T4 DNA ligase, HIV nucleic acid hybridizes with padlock DNA to form the template (circular ssDNA) for the RCA reaction. The walking strand generated during the RCA reaction moves along the 3D track, resulting in recovered fluorescence signals. The integration of these two amplification methods results in a detection limit of 1.46 fM, indicating great potential of this method for biomedical research and clinical applications. The integration of RCA and DNA walkers can also be achieved on the electrochemical platform. Cai et al. [109] developed a dual-signal amplification electrochemical biosensor based on DNA nanoflowers, DNA walkers, and aptamers. By optimizing experimental conditions, this device achieved a detection limit of 9 CFU/mL for Staphylococcus aureus and can be used in drinking water and diluted honey samples.

### 3.2. DMMs for Multiplex Detection of Biomarkers

**Figure 6:**
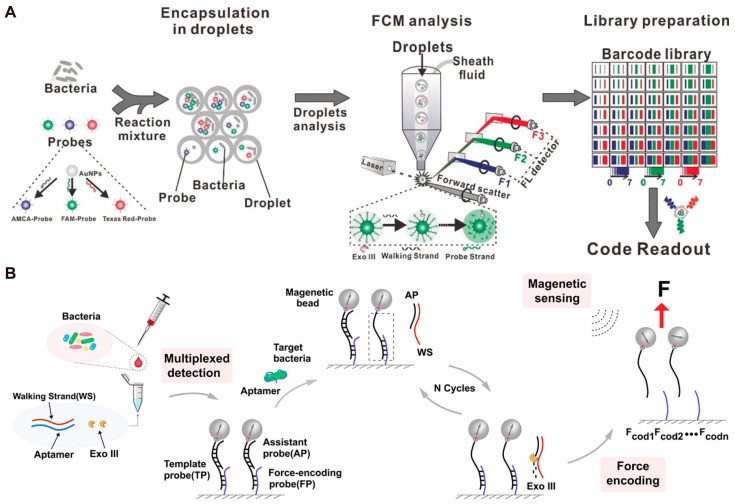
Examples of multiple DMMs simultaneously for multiplex detection. (**A**) Schematic image of SDwalker-Drop for the multiplex bacterial phenotype detection. Reprinted with permission from [34]. Copyright 2019, Wiley-VCH Verlag GmbH & Co. KgaA, Weinheim, Germany. (**B**) Schematic image of force-encoding DMMs for detecting multiplexed bacteria in blood. Reprinted with permission from [113]. Copyright 2024, American Chemical Society.

In recent years, simultaneous detection of multiple analytes has become increasingly important in biomedical applications because single biomarker detection cannot achieve accurate diagnosis of diseases [110]. DNA computing strategies can be integrated into DMMs to identify different analytes and generate multiplexed output signals [111,112]. Xiao et al. [34] developed the stochastic DNA walker in droplets (SDwalker-Drop) for high-throughput detection of bacteria. The binding of bacteria can initiate the DNA walker to generate fluorescent signal. Subsequently, they encapsulated multiple SDwalkers and bacteria in droplets in precise proportions and quantities and used flow cytometry to detect fluorescence intensity in individual droplets (Figure 6A). By embedding intensity-encoded barcodes into a sequence of color-multiplexed barcodes, the detection and identification of 20 different bacterial phenotypes was realized using this strategy. Recently, Sun et al. [113] integrated a force-based encoding strategy with DMMs. As shown in Figure 6B, after bacteria binding to the aptamer, a complementary walking strand is released and then reacts with the tracking strand that has been immobilized onto an aldehyde-terminated substrate, resulting in the release of template probes (TP). The binding forces of TP-force encoding probes (FP) are different when different bacteria are recognized, leading to distinguishable residual magnetic signal. Using this strategy, they achieved multiplex detection of various pathogens in blood samples with no washing step.

DMMs can be employed for multiplex detection inside cells. In 2013, Modi et al. [114] achieved the simultaneous operation of multiple DMMs within the same cell for the first time. Two types of DMMs are used for the simultaneous monitoring of pH changes in two different endocytic pathways. The first DMM, which is a DNA tweezer, contains the binding site of an engineered protein and can be internalized via the furin pathway. The DNA tweezer displays distinct fluorescence signals depending on whether it is open or closed. Another nanodevice is a dsDNA probe containing an i-motif sequence, which is chemically linked to transferrin (TF) and can be internalized into cells through the transferrin receptor pathway. After entering the same cell through different pathways, the two DMMs were colocalized in early endosomes and then proceeded to late endosomes and recycling endosomes, respectively [115]. Because they function autonomously within the cell, these DMMs can simultaneously monitor the pH levels of diverse endocytic pathways within the same cell.

The above-mentioned strategies all use multiple nanodevices to achieve simultaneous detection of multiple targets. Considering the cost, simplicity and practical application, it is more favorable to integrate multiple signal-responsive modules into the same DMM [116]. Taking the dual-functional DNA tweezer (DFDT) developed by Yang et al. as an example [117], the targets miRNA-21 and mucin 1 (MUC1) are converted into two distinct fuel strands via their complementary sequences and aptamer, respectively. The two fuel strands hybridize with the DNA tweezer, inducing conformational change and fluorescence quenching. Both targets can be detected simultaneously because each target induces fluorescence quenching in different channels. The DFDT-based strategy achieved an LOD of 32 fM for miRNA-21 and an LOD of 8.5 pM for MUC1. In the same year, Chang et al. [118] constructed a classified cargo-discharge DNA robot for ultrasensitive electrochemical detection of miRNA-155 and miRNA-21. Upon hybridization with the target, the DNA robots rapidly move across the electrode surface. Subsequently, DNA fragments labeled with methylene blue (MB) or ferrocene (Fc) on the electrode surface are selectively released upon cleavage by Nt.BbvCI. The release of different RNA targets leads to the decrease in electrochemical signal at different voltages. This DNA robot can detect miRNA-155 and miRNA-21 simultaneously with good sensitivity (LOD: 42.7 aM and 51.1 aM, respectively).

**Figure 7:**
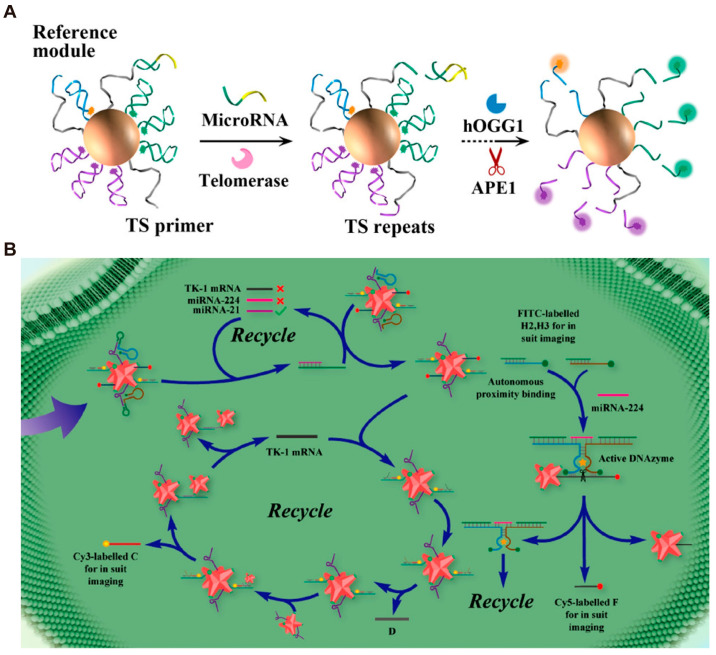
Examples of single DMM integrated multiple responsive modules for multiplex detection. (**A**) Schematic image of working process of all-in-one synchronized DNA nanodevices. Reprinted with permission from [119]. Copyright 2019, American Chemical Society. (**B**) Schematic images of diagnosis and treatment process of the Au/Pd ONP-DNA nanomachines. Reprinted with permission from [120]. Copyright 2021, American Chemical Society.

Embedding multiple response modules into a single device can achieve simultaneous detection of multiple biomarkers within cells. Xue et al. [119] developed a multifunctional DNA walker to monitor microRNA-21 and the expression levels of telomerase within cells. Two sets of probe modules and a reference module are anchored to the same AuNPs (Figure 7A). The two probes can respond to two different targets severally and generate distinct fluorescent signals. Additionally, the enzymes required for target recognition can also cleave the reference module, producing corresponding fluorescence signals. This process serves as a self-reference for the changes in enzyme activity in different cells. Ultimately, this DMM was successfully applied for synchronous multi-channel imaging inside living cells. Cai et al. [120] integrated multiple logic operation systems onto a single nanoparticle to construct Au/Pd octopus nanoparticles–DNA nanomachines (Au/Pd ONP-DNA nanomachines). Only when the primary biomarker (miRNA-21) is present can the two secondary biomarkers (miRNA-224 and TK-1 mRNA) be detected (Figure 7B). When all three targets are present (input as (1,1,1), output as (1,1)), the output segment is subjected to an 808 nm laser for photothermal and photodynamic therapy of tumors. Through the construction of three AND logic gates, this system enables the fully automated diagnosis and precise treatment of tumor cells. In addition to intracellular detection, DMMs can also be used to detect interactions between cells and their microenvironment. Shahhosseini et al. [121] integrated DNA origami-based DMMs into a microfluidic platform and realized multiplex sensing on the cell surface. This platform can simultaneously detect two specific molecules and monitor the cell–microenvironment interaction at subcellular resolution.

### 3.3. DMMs for Real-Time Monitoring of Biomarkers

**Figure 8:**
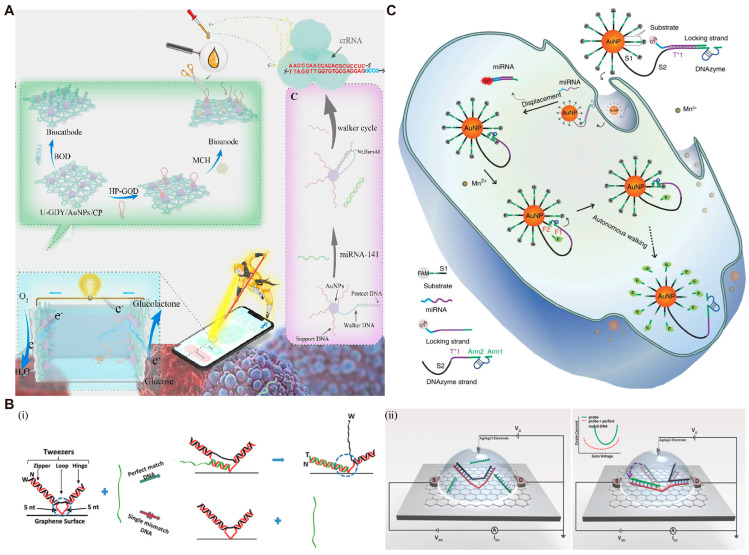
Examples of DMMs for real-time detection. (**A**) Construction and work principle of self-powered biosensor. Reprinted with permission from [124]. Copyright 2023, Elsevier B.V. (**B**) Schematic images of FET sensor with DT probes. Reprinted with permission from [125]. Copyright 2018, WILEY-VCH Verlag GmbH & Co. KGaA, Weinheim, Germany. (**C**) Schematic image of 3D DNA walker for specific miRNA imaging. Reprinted with permission from [126]. Copyright 2017, Springer Nature.

Real-time monitoring capability is essential for the application of biosensors in accurate disease diagnosis and disease recurrence monitoring [122,123]. Xu et al. [124] developed a 3D DNA walker-mediated self-powered biosensor for rapid and real-time analysis of miRNA. Upon the appearance of the target miRNA-141, the DNA walker is initiated to release numerous single strands. These single strands hybridize with crRNA, activating the nonspecific anti-cutting capabilities of CRISPR/Cas12a (Figure 8A). The cleavage of the specific site of the capture probe (HP) by CRISPR/Cas12a leads to the release of glucose oxidase, which can further generate electrical signal. This design not only achieves excellent sensitivity, but also allows real-time reading of detection results through a smartphone application. Field effect transistor (FET) is also used for sensitive and real-time detection of analytes. Hwang et al. [125] combined FETs with DNA tweezers (DT) to detect single nucleotide polymorphism (SNP). Through π−π stacking and amine-amide bonding, DT probes are horizontally anchored to the graphene surface (Figure 8B). Upon perfect matching, the strand displacement reaction occurs between the target strands and DT, resulting in the elongation of the DNA sequence at the charged accumulation region. At this point, the probe moves closer to the graphene surface, generating a stronger electrical signal. The strand displacement reaction cannot happen in the presence of mismatched target strands, so that no electrical signal change can be observed. This sensor can be fabricated into a small and portable chip, enabling real-time transmission of the generated electrochemical signal to personal electronic devices.

DMMs can be also implemented for real-time in situ imaging of biomarkers in living cells. In 2009, Modi et al. [127] pioneered the application of DMMs for live-cell imaging by designing an i-motif-based DNA tweezer. At pH 5, the i-motif structures are formed, and DNA tweezers are closed, resulting in the FRET signal between Alex-488 and Alex-647. With a transferrin modification, this device can be efficiently taken up by cells into endosomes via transferrin receptor-mediated uptake for real-time imaging of pH changes. Other DMMs for real-time imaging in living cells were subsequently reported [114,128,129,130,131,132], indicating the potential of DMMs in this field. DNA walkers have also been utilized for intracellular analyte imaging due to the high signal amplification efficiency. In 2016, Peng et al. [126] constructed the first 3D DNA walker capable of operating inside living cells. After being activated by the intracellular target miRNA, the released DNA walker cleaves the substrate strand to recover the fluorescence (Figure 8C). Using design, miR-10b in human breast cancer cells (MDA-MB-231 cell line) were effectively quantified and imaged. The 3D DNA walker can also be utilized for in vivo imaging. Yin et al. [133] used the DNA walker for real-time imaging of amyloid-beta peptide oligomers (AβO) in live cells and in vivo. The entire detection process of AβO is autonomous and continuous, without the need of external fuel strands and proteases. Therefore, this design allows for both the quantitative detection of AβO in vitro (with a detection limit of 22.3 pM) and real-time imaging of AβO in live cells and in vivo. Additionally, based on in vivo imaging results in mice, the DNA walker can effectively distinguish between Alzheimer’s disease mice and wild-type mice. It should be noted that DMMs showed no obvious cytotoxicity or side effects on living cells according to the results in the above-mentioned studies, benefiting from the inherent biocompatibility of DNA.

### 3.4. DMMs for Spatial Recognition Detection of Biomarkers

**Figure 9:**
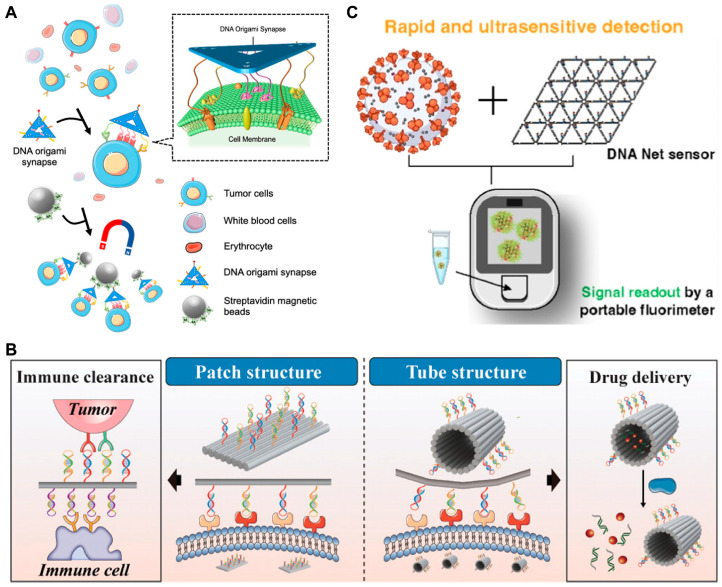
Examples of DMMs for spatial recognition detection. (**A**) Schematic of DNA nanoarrays for precise recognition of target cells. Reprinted with permission from [134]. Copyright 2023, American Chemical Society. (**B**) Schematic images of multivalent aptamer-based DMMs for recognizing proteins. Reprinted with permission from [135]. Copyright 2024, American Chemical Society. (**C**) Schematic image of DNA nets for viral capture and reading/inhibition. Reprinted with permission from [137]. Copyright 2023, American Chemical Society.

Membrane proteins on the cell surface are important biomarkers for disease diagnosis and treatment. Consequently, the recognition and detection of membrane proteins has become a hot research topic in recent years. However, some proteins are under-expressed and disordered on the cell surface, which significantly affects the accurate recognition of membrane proteins and thus limits the exploration of related signaling pathways. To solve this problem, Mao et al. [134] constructed a nanoarray based on multivalent aptamers and DNA origami to recognize low-affinity antigen-specific cells. As shown in Figure 9A, by adjusting the valence and spacing of aptamers (Apt-EpCAM) and adding aptamers of auxiliary molecules (Apt-EGFR and Apt-HER2), they constructed a nanotopology structure that matched the spatial distribution of membrane proteins (epithelial cell adhesion molecule, EpCAM). The nanotopology-based nanoarray showed significantly enhanced binding affinity and specificity and were successfully used for the detection of low-affinity antigen in heterogeneous circulating tumor cells (CTCs). Recently, Hu et al. [135] used a similar strategy to construct multivalent aptamer-based DMMs for recognizing proteins on the surface of tumor cells (Figure 9B). By adjusting the aptamer species, valence, and spatial location, they found that the multi-heteroreceptor-mediated recognition not only improved the specificity of the device in recognizing tumor cells, but also greatly enhanced cellular uptake. Unlike previous studies, which were limited to recognition and detection, their DMM can be also used for targeted drug delivery, modulated ell-cell interactions, and effective immune clearance.

Apart from cells, DMMs can also be used to encapsulate entire virus particles through precise spatial recognition patterns. In 2020, Kwon et al. [136] integrated aptamers with DMMs to construct DNA stars for viral sensing and inhibition. The spatial distribution of aptamers targeting the envelope protein domain III (ED3) on the star-shaped DNA structure precisely matches the distribution of ED3 on the dengue (DENV) virus surface. The binding between aptamer and virus can stretch the nanostar to adapt to the shape of virus and the distribution pattern of the target protein on virus surface. They found that compared to DNA six-point stars and DNA seven-point stars, DNA five-point stars exhibited higher detection sensitivity, indicating the crucial role of precise spatial recognition in enhancing detection sensitivity. Later, Chauhan et al. [137] employed a similar strategy to construct DNA nets for capturing the SARS-CoV-2 virus (Figure 9C). Aptamers decorated on the DNA nets can form a series of trimer clusters capable of matching the trimeric spikes on virus surface and inducing multivalent interactions. They demonstrated that compared to simple nucleic acid probes or junction structure, DNA net structures exhibit the best sensitivity. Benefiting from the accurate spatial recognition patterns, the strategy can be used not only for rapid, simple, sensitive and room-temperature compatible COVID-19 detection, but also for inhibiting SARS-CoV-2 infection.

### 3.5. DMMs for Single-Molecule Detection of Biomarkers

**Figure 10:**
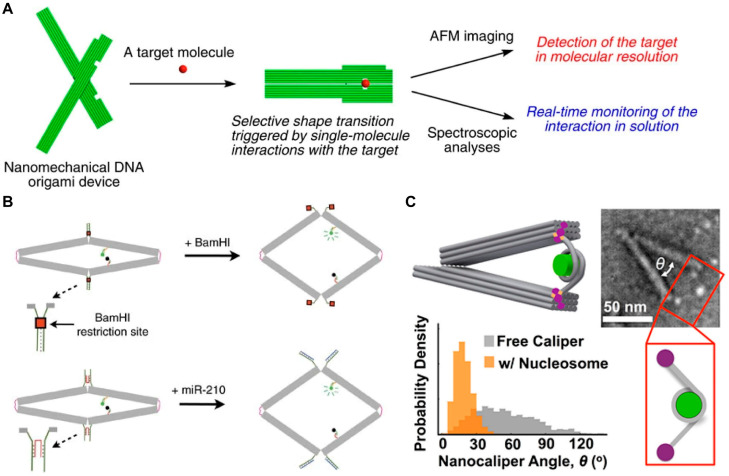
Examples of DMMs for single-molecule detection. (**A**) Schematic of the DNA plier for detection of various targets. Reprinted with permission from [138]. Copyright 2011, Springer Nature. (**B**) Schematic illustration of the dynamic DNA origami devices for single-molecule detection. Reprinted with permission from [139]. Copyright 2016, Springer Nature. (**C**) Schematic image of the DNA nanocaliper for investigating the disassembly of nucleosome structures. Reprinted with permission from [137]. Copyright 2016, American Chemical Society.

Some DNA origami-based DMMs can be observed with single-molecule resolution by atomic force microscopy (AFM) or electron microscopy, making them suitable for single-molecule detection of biomarkers. Kuzuya et al. [138] developed a functional nanomechanical DNA plier for single-molecule detection of various biomolecules, including streptavidin, IgG, Ag^+^, and miRNA (Figure 10A). They connected two approximately 170 nm long levers at a fulcrum, with respective ligands attached to each lever. In the presence of the target, binding between the target and ligands causes the DNA plier to close. The shape change in the DNA origami pliers can be observed using AFM, thus enabling intuitive detection of biomolecules at the single-molecule level. Later, Ke et al. [139] constructed dynamic DNA origami devices for single-molecule detection (Figure 10B). The top of the rhombus-shaped DNA origami was locked in a closed state by different mechanisms. When target molecules appeared, the locking mechanism was released, allowing the origami to return to its open state. The structural change in DMMs can be also measured using fluorescence intensity by modifying dye/quencher pair on the rhombus structure. Finally, this design was used as a “spring-loaded” nanosensor for ions, RNAs, or enzymes. Meanwhile, unlike the dynamic origami structures constructed in previous studies, this DNA origami nanomotor can propagate distance changes initiated on one half of the structure (“driver”) to the other half (“mirror”), thereby achieving remote control. This remote control does not affect the availability or reactivity of the substrate. With the increasing flexibility in DNA origami design, Wang et al. [140] introduced DNAzymes into DNA origami. They constructed DMMs capable of responding to Zn^2+^ and Pb^2+^, achieving multiplex detection at the single-molecule level.

DMMs can translate interactions between biomolecules into changes in their own structure, making them commonly used for analyzing molecular interactions at the single-molecule level, thereby revealing molecular mechanisms of certain biological processes. Funke et al. [141] constructed tweezer-shaped DNA origami to measure interactions between nucleosomes. Two nucleosomes were tethered to the two arms of DNA origami-based force spectrometer via DNA hybridization. They measured the distance between the two nucleosomes at subnanometer resolution using single-particle electron microscopy. They concluded that the relative orientation of nucleosomes did not affect their interaction, but acetylation of histone H4 or removal of histone tails reduced the strength of their interaction. In the same year, Le et al. [142] employed a similar design to investigate the disassembly of nucleosome structures (Figure 10C). They constructed a DNA origami-based nanocaliper and attached nucleosomes to the ends of two caliper arms. The study demonstrated that the angle of the nanocarrier accurately reflects the distance between the two ends of the nucleosome, serving as a sensitive indicator of nucleosome disassembly. Therefore, researchers can determine the binding of transcription factors to target sites within nucleosomes based on this angle value, laying the foundation for subsequent studies on DNA–protein interactions and chromatin conformational changes.

## 4. Conclusions and Outlook

Propelled by the development of DNA nanotechnology, DMMs have become more sophisticated in their structures [46,58,60] and mechanical functions [45,50,51]. In this review, we classified DMMs by their types of stimuli, including DNA fuels, enzymes, electric fields, light and other stimuli. We summarized the mechanisms behind each type of stimulus that provides energy for DMMs. Subsequently, we described the latest advancements of DMMs in biosensing applications. DMMs possess significant advantages such as strong programmability, nanoscale controllability, and design flexibility. Additionally, in recent years, numerous recognition elements and signal transduction methods have been integrated into DMMs. Given these excellent characteristics, DMMs have found wide applications in amplified, multiplex, real-time, special recognition, and single molecule biosensing and bioimaging.

However, DMMs still face crucial challenges. Firstly, the stability of DMMs needs to be further improved. DNA is easily degraded in the body. The integrity of the structure is necessary for the DMMs to work in complex bioenvironment but degradation by enzymes can render DMMs inactive. Although some chemical modifications have been reported to resist degradation [143], these additional modifications can significantly affect the thermodynamic properties of DNA, thereby compromising the controllability and predictability of DMMs. Secondly, with multiple repeated operations of DMMs, their operating efficiency will gradually decrease. This may be due to the unexpected structure changes in DMMs caused by reaction leak during operation that leads to the deactivation of DMMs. Thirdly, it is worth considering how to reduce the cost of DMMs. With the development of solid-phase DNA synthesis technology, the cost of DNA synthesis has been greatly reduced, but it is still expensive compared to the synthesis of small molecules. Additionally, it is an important challenge to achieve mass production of DMMs. Finally, although many studies have demonstrated that DMMs exhibit no significant toxicity when applied intracellularly or in vivo [144], there are still concerns of biosafety risks. As the core component of DMMs, DNA may be integrated into the genome of cell through genetic recombination or effect the expression of mRNA. Improper genetic recombination or undesirable gene silencing could alter cell function or even lead to cellular carcinogenesis. Moreover, most relevant studies have been conducted in mouse models with experimental periods typically lasting 3–6 months, which falls short of the long-term biosecurity assessment standards required for clinical applications.

In the future, advancements in the controllability, stability, and operational efficiency of DMMs can be expected, and the cost of constructing DMM will be significantly reduced. Furthermore, we anticipate that the in vivo toxicology of DMMs will be thoroughly investigated in future studies. As the design of DMMs becomes more biocompatible, sustainable, intelligent, and portable, DMMs will find broad application prospects in point-of-care testing and clinical diagnostics. Additionally, DMMs may be used to replace damaged natural molecular machines, provide cells with additional functions, and act as important functional modules in synthetic artificial cells, ultimately achieving a more precise regulation or simulation of biological systems.

## Data Availability

Not applicable.
